# Notes and News

**Published:** 1896-11-28

**Authors:** 


### NOTES AND NEWS.

(COLLECTED AND COMMUNICATED.)

ST. GEORGE'S HOSPITAL desires to found a memorial to Jenner, who was a student there. A meeting to decide what form the memorial is to take will be held on December 7th, with Sir Joseph Lister in the chair.

WE are informed that the Lord Mayor has consented to take the chair at the annual dinner of the Metropolitan Hospital to be held at the Whitehall Rooms on Thursday, March 18th next.

IN connection with the Dongola Expedition military honours have been conferred on Brigade-Surgeon Lieut.-Colonel Gallwey, who is made Companion of the Bath; and on Surgeon-Major Hunter and Surgeon-Captain Penton, both created Companions of the Distinguished Service Order; whilst Surgeon-Major Sloggett is promoted to be Lieutenant-Colonel.

IN view of the present inadequate provision for the sick at Portsmouth, a new hospital for from three hundred to three hundred and fifty beds is contemplated. The Chief Magistrate, Mr. Couzens, expresses his intention of doing all in his power to further the project. The site of the present hospital is considered good, and the scheme may therefore take the form of an extension, if this admits of a thoroughly up-to-date institution being secured for Portsmouth.

THE new Research Laboratory of the Royal College of Physicians of Edinburgh has been formally opened, and work is now being carried on there. No expense has been spared to secure an institution worthy of the object for which it has been established, and to render it complete in every way. The special objects kept in view are adequate appliances for research in bacteriology and histology and in chemical and experimental physiology and pathology, to provide for the examination of morbid specimens for medical men, and for ordinary microscopic photographic work. Suitable provision for the preparation of curative anti-toxins is also a feature.

HALF A MILLION working men signed a petition to the Home Secretary against the granting of a licence to experiment on living animals at the Pasteur Institute at Chelsea. The Home Secretary replied that he had not yet received an application for registration from the Institute, and states that he does not think it necessary to receive another deputation.

THE long services of Mr. Abrams as secretary of the Central London Ophthalmic Hospital have been recognised by the committee by the presentation of a silver cup, bearing the following inscription: "Presented to Wm. Abrams, Esq., by the committee of the Central London Ophthalmic Hospital, on his retirement from the post of secretary, as a mark of their esteem, 1896."

THE Sir Augustus Harris Memorial Fund has now reached upwards of £2,000. It is proposed to devote it to a hospital for a ward to be named after Sir Augustus Harris, and to be exclusively kept for sufferers who have been connected with the theatrical or musical professions. It is also proposed to erect, somewhere in the neighbourhood of Covent Garden, a suitable memorial drinking fountain.

THE Chairman of St. Mary's Hospital, Manchester, has replied to the many letters which have appeared in the local press, respecting the hospital and the David Lewis trustees. He devotes himself to explaining that the main objections of his committee to the amalgamation scheme are that the site proposed is one which had been previously rejected by St. Mary's as unsuitable, and that the Owens College would have too large a hold upon it under the proposed arrangement, and that all separate individuality as an institution would be lost to his hospital.

A CONFERENCE of leprologists and delegates of various Governments will be held at Berlin in October, 1897, to discuss the slow but sure increase of leprosy and the preventive measures to oppose the plague. The conference was fixed for March, 1897, but the scientifical expedition of Professor Rob. Koch to South Africa (for the British Government) has necessitated the postponement of the date. The members of the Organizing Committee are Mm. Armauer Hansen (Bergen in Norway), Rob. Koch, and O. Lassar (Berlin), Edv. Ehlers (Copenhagen) as secretary.

MR. BANCROFT held the first of his promised readings on behalf of the hospitals on Monday last at the Queen's Hall. The subject chosen was Dickens' "Christmas Carol," and the object for which the reading was held was the cancer wards of the Middlesex Hospital. The novel entertainment was thoroughly appreciated by the large audience assembled. Mr. Bancroft's treatment of his subject was masterly; his arrangement of the story for verbal reproduction was so complete that all the best features were brought together and woven into a continuous whole. It is difficult to say whether the humorous or the pathetic passages, so closely following one another in all Dickens' works, were the more artistically rendered. We are glad to hear that Mr. Bancroft has already accepted an invitation to appear in the Sheldonian Theatre, Oxford, on behalf of the Radcliffe Infirmary, and that later he is going to honour Cambridge with a visit.

